# *Eurosurveillance* reviewers in 2016

**DOI:** 10.2807/1560-7917.ES.2017.22.1.30432

**Published:** 2017-01-05

**Authors:** Eurosurveillance editorial team

**Affiliations:** 1 European Centre for Disease Prevention and Control (ECDC), Stockholm, Sweden

The editorial team is grateful to all the experts who dedicated their time to peer-review our articles over the past 12 months. We wish to thank them and all those whose names are not listed below but gave helpful advice and support. We apologise to any reviewers whose names we may have missed.

Our reviewers in 2016 were:

Preben Aavitsland; Rachid Aaziz; Florence Abravanel; Cornelia Adlhoch; Franz Allerberger; Maria an der Heiden; Sören Andersson; Marta Andrés Miguel; Nick Andrews; Fabrizio Anniballi; Ozgur Araz; Maris Arcilla; Albena Arnaudova; Barry Atkinson; Jean-Luc Bailly; Meghan Baker; Koye Balogun; Elizabeth Bancroft; Norbert Bannert; Anne-Laure Bañuls; Ian Barr; Nicholas Beeching; Edward Belongia; Peter Ben Embarek; Anne Berger-Carbonne; Björn Berglund; Vincent Béringue; Olaf Berke; Kathy Bernard; Antoine Berry; Zeno Bisoffi; Par Bjelkmar; Pierre-Yves Boelle; Shelly Bolotin; Magnus Boman; Francesco Bonfante; Isabelle Bonmarin; Marc Bonten; Stefan Borgmann; Scott Bowden; Walter Boyce; Heather Bradley; Marieta Braks; Arne Brantsæter; Viviane Bremer; Sharon Brookes; Tim Brooks; Lisa Brouwers; Kevin Brown; Mathias Bruyand; Mia Brytting; Udo Buchholz; Silke Buda; Herbert Budka; Daniel Cadar; Shona Cairns; Mattia Calzolari; Romina Camilli; Helen Campbell; Rafael Canton; João André Carriço; Joan Casey; Alessandro Cassini; Nadim Cassir; Jesús Castilla; Dominique Caugant; Vicki Chalker; Meera Chand; Yves Charpak; Remi Charrel; Daniel Chemtob; Mark Chen; Allen Cheng; Gerardo Chowell; Bruno Ciancio; Niel Constantine; Victor Corman; Benjamin Cowling; Peter Coyle; Natasha Crowcroft; Maria Teresa Cuevas; François Dabis; Harry Dalton; Stephanie Dancer; Niklas Danielsson; Mark Danta; Nadav Davidovitch; Helena de Carvalho Gomes; Birgitta de Jong; Jesús de Pedro Cuesta; Koen De Schrijver; Gaston De Serres; Henriette De Valk; Anouk Decors; Tarik Derrough; Jean-Claude Desenclos; Liselotte Diaz Högberg; Sabine Diedrich; Mercedes Diez; David Dowdy; Jan Felix Drexler; Christian Drosten; Tarun Dua; Mariette F. Ducatez; Myrielle Dupont-Rouzeyrol; Boris Dzelalija; Paul Effler; Alex Ensminger; Susanna Esposito; Steen Ethelberg; Iro Evlampidou; David Eyre; Daniel Faensen; Dennis Falzon; Amanda Faulkner; Helmut Fickenscher; Nigel Field; James Fielding; Daniel Fierer; Antonietta Filia; Thea Fischer; David Fisman; Ross Fitzgerald; Julia Fitzner; Brendan Flannery; Ivo Foppa; Claudia Fortuna; Christina Frank; Eelco Franz; Derek Freedman; Alexander Friedrich; Ingrid Friesema; Hans-Peter Fuehrer; Katie Fullerton; Cristina Furtado; Carlo Gagliotti; Montserrat Gállego; Manuel García Cenoz; Luis García-Comas; Giuliano Garofolo; Anthony Gatt; Philippe Gautret; Peter Gerner-Smidt; Johan Giesecke; Pere Godoy; Magnus Gottfredsson; Stephan Göttig; Hannelore Gotz; Luigi Gradoni; Kathie Grant; Donato Greco; Michel Grignon; Olaf Gudlaugsson; Volker Gurtler; Brian Gushulak; Jean-Paul Guthmann; Bernardo Rafael Guzman Herrador; Karin Haar; Walter Haas; Christos Hadjichristodoulou; Susan Hahne; Ágnes Hajdu; Sebastian Haller; Pia Hardelid; Timm Harder; Heli Harvala; Henrik Hasman; Angelos Hatzakis; Christopher D. Heaney; Edou R. Heddema; Wiebke Hellenbrand; Greet Hendrickx; Bjorn Herrmann; David Heymann; Markus Hilty; Martin Hoenigl; Christiane Höller; Emma Hollis; Vahur Hollo; Anders Holst; Vivian Hope; Judit Krisztina Horvath; G Hrčkova; Yhu-Chering Huang; Judith Hübschen; Yvan Hutin; Benedikt Huttner; Daniela Huzly; Tetsuro Ikegami; Silviu Ionescu; Giuseppe Ippolito; Masako Iwanaga; Aftab Jasir; Johanna Jefferies; Cecilia Jernberg; Ana Jeroncic; Kari Johansen; Joerg Jores; Aleksandra Jovanovic Galovic; Dennis Junqueira; Bernard Kaic; Rolf Kaiser; Anu Kantele; Olen M Kew; Philip Kiely; Lisa King; Martyn Kirk; Esther Kissling; Irena Klavs; Silke Klee; Don Klinkenberg; Hilde Kløvstad; Jan Kluytmans; Mira Kojouharova; Saara Kotila; Natalia Kozak-Muiznieks; Alenka Kraigher; Piotr Kramarz; Gerard Krause; Tyra Krause; Peter Kreidl; Karen A. Krogfelt; Adam Kucharski; Ed Kuijper; Martin Kulldorff; Jason C. Kwong; Guy La Ruche; Angie Lackenby; Shamez Ladhani; Philippe Lagacé-Wiens; Theresa Lamagni; Miranda Langendam; Karine Laroucau; Daniel Lavanchy; Jeffrey Lazarus; Simon Le Hello; Jason J Leblanc; Isabelle Leparc-Goffart; Isabel Leroux Roels; Justin Lessler; Daniel Lévy-Bruhl; Sébastien Lhomme; Troels Lillebaek; Bruno Lina; Marc Lipsitch; Irina Ljungqvist; Pierluigi Lopalco; Maria Angeles Lopaz; Rosa Lopez-Gigosos; Rachel Lowe; Claudia Lucarelli; Alexander Lukashev; Åke Lundkvist; Jennifer MacLachlan; Jean-Yves Madec; Gkikas Magiorkinis; Henri-Pierre Mallet; Helena Maltezou; Caterina Mammina; Jean-Michel Mansuy; Jean-Claude Manuguerra; Ulrich Marcus; Clara Marín; Henju Marjuki; Laurence Marrama; Emily Martin; Susan Maslanka; Jean Mbisa; Peter McIntyre; Jim McMenamin; Mary Meehan; Adam Meijer; Angeliki Melidou; Ella Mendelson; Stefano Merler; Kevin Messacar; Elisabeth Meyer; Peter Michielsen; Nkuchia M'ikanatha; Vivi Miriagou; Kåre Mølbak; Thomas Mollet; Carmen Montaño-Remacha; Jacob Moran-Gilad; Simon More; Alain Moren; Robert Moss; Joël Mossong; Varvara Mouchtouri; Lapo Mughini-Gras; Miguel Mulders; Matthew Muller; Marcel Müller; Didier Musso; Thierry Naas; Celine A Nadon; Monika Naus; Richard Neher; Matthias Niedrig; Sandra Niendorf; Harold Noel; Paulo Jorge Nogueira; Hanna Nohynek; Hanne Nokleby; Teymur Noori; Daan Notermans; Pierre Nouvellet; Norbert Nowotny; Elina Numminen; Sophie Octavia; Ruth Offergeld; Justin O'Hagan; Åke Örtqvist; Masaki Ota; Makoto Ozawa; Takis Panagiotopoulos; Marcus Panning; Anna Papa; Vassiliki Papaevangelou; Dimitrios Paraskevis; Antonio Parisi; Gabriel Parra; Joseph Peiris; Luisa Peixe; Tatjana Pekmezovic; Pasi Penttinen; Kiran Perkins; Lyle Petersen; Efi Petinaki; Helen Petousis-harris; Martin Petric; Günter Pfaff; Dina Pfeifer; Yvonne Pfeifer; Anastasia Pharris; Mathieu Picardeau; Denis Piérard; Johann Pitout; Diamantis Plachouras; Laurent Poirel; Michel-Robert Popoff; Thomas Pottage; Kevin Pottie; Edoardo Pozio; Anne Presanis; Elisabeth Presterl; Andrea Pugliese; Wolfgang Rabsch; Jayna Raghwani; Mario Ramirez; Brian Raphael; Rod Ratcliff; Gili Regev-Yochav; Chantal Reusken; Giovanni Rezza; Anders Rhod Larsen; Thomas V. Riley; Shmuel Rishpon; Caterina Rizzo; Beatriz Rojo-Bezares; Constanze Rossmann; Paul Rota; Adam Roth; Maja Rupnik; Etienne Ruppe; Werner Ruppitsch; Kristi Rüütel; Tomoya Saito; Juan-Carlos Saiz; Stefania Salmaso; Dan Salmon; Lamia Samad; Örjan Samuelsen; Anita Sands; Monica Sañé Schepisi; Hugo Sax; Gianpaolo Scalia-Tomba; gaia scavia; Robert Scharff; Aaron Scherer; Patricia Schlagenhauf; Clara Schlaich; Axel Schmidt; Jonas Schmidt-Chanasit; Henrik Carl Schønheyder; Isabelle Schuffenecker; Lavinia Schuler-Faccini; Richard Selik; Ettore Severi; David Shay; Lester Shulman; Andrew Simpson; Andreas Sing; Vitali Sintchenko; Caroline Six; Lars Skog; Robert Skov; Danuta Skowronski; Werner Slenczka; Pierre Smeesters; David Smith; Heidi Soeters; Ruben Solano; Vincent Soriano; Mark Sotir; Erica Spackman; Gianfranco Spiteri; Danica Stanekova; Paola Stefanelli; Chen Stein-Zamir; Roger Stephan; Marc Struelens; Johan Struwe; Bertrand Sudre; Philippe Sudre; Carl Suetens; Jonathan Suk; Sheena Sullivan; Martin Sundqvist; Edit Szegedi; Silvio Tafuri; Johanna Takkinen; Yi-Wei Tang; Dennis Tappe; Masato Tashiro; Anders Tegnell; Rhys Thomas; Lelia Thornton; John Threlfall; Sera Tort; Katy Town; Stephen Tristram; Sotirios Tsiodras; Marta Valenciano; Janko van Beek; Birgit van Benthem; Wim van Bortel; Marita van de Laar; Marianne van der Sande; Marieke J. van der Werf; Steven Van Gucht; Albert van Hoek; Maria Van Kerkhove; Alies van Lier; Wilfrid Van Pelt; Ivo van Walle; Carmen Varela Santos; Istvan Varkonyi; Alkiviadis Vatopoulos; Inga Velicko; Harry Vennema; Jan Vinjé; Anne-Catherine Viso; Tanja Vollmer; Christina von Hunolstein; Rengina Vorou; Jiri Wagenaar; David Walker; Timothy Walker; Anders Wallensten; Jacco Wallinga; Hester Ward; Bryna Warshawsky; Pierre Wattiau; Todd Weber; Geoffrey Weinberg; Guido Werner; Katarina Westling; Ole Wichmann; Micael Widerström; Miriam Wiese-Posselt; Hendrik Wilking; Christopher Williams; Carl-Heinz Wirsing von König; James Wood; Hui-Ling Yen; Maria Zambon; Herve Zeller; Loukia Zerva; Ruth Zimmermann; Walter Zingg.

